# Comparison of Different Rabbit Anti-Thymocyte Globulin Formulations in the Prophylaxis of Graft-Versus-Host Disease: A Systematic Review

**DOI:** 10.3390/jcm12175449

**Published:** 2023-08-22

**Authors:** Jarosław Dybko, Ugo Giordano, Justyna Pilch, Jakub Mizera, Artur Borkowski, Monika Mordak-Domagała

**Affiliations:** 1 Lower Silesia Centre for Oncology, Pulmonology and Hematology in Wrocław, 53-439 Wroclaw, Poland; dybko@post.pl (J.D.); mordak@dctk.wroc.pl (M.M.-D.); 2 University Clinical Hospital in Wroclaw, Wroclaw Medical University, 50-556 Wroclaw, Poland; 3 Faculty of Medicine, Wroclaw Medical University, 50-367 Wroclaw, Poland; justyna.pilch@student.umw.edu.pl (J.P.); jakub.mizera@student.umw.edu.pl (J.M.); 4 Department of Nuclear Medicine and Endocrine Oncology, M. Sklodowska-Curie National Research Institute of Oncology Gliwice Branch, 44-102 Gliwice, Poland; artur.borkowski.md@gmail.com

**Keywords:** anti-thymocyte globulin, acute graft-versus-host disease, chronic graft-versus-host disease, allogeneic stem cell transplantation, thymoglobuline, grafalon

## Abstract

Allogeneic hematopoietic stem cell transplantation (allo-HCT) is a potentially curative treatment modality, frequently used for patients suffering from haematological malignancies. In the last two decades, there have been multiple randomised controlled trials (RCTs), review articles, and meta-analyses addressing the efficacy of rabbit anti-thymocyte globulin (r-ATG) as a graft-versus-host disease (GvHD) prophylaxis. Nevertheless, only a few aimed to compare the effectiveness of different r-ATG formulations. Since the last article we retrieved comparing different r-ATGs in GvHD prophylaxis dates back to 2017, we performed a systematic literature review of articles published since 2017 to this day, utilising PubMed, Scopus, Cochrane, and MEDLINE, with the main endpoints being prophylaxis of acute GvHD (aGvHD) and chronic GvHD (cGvHD). We subjected to scrutiny a total of five studies, of which four compared the differences between Thymoglobulin (ATG-T) and Grafalon (ATG-G), and one discussed the impact of ATG-T dose. Overall, cGvHD, aGvHD grades II–IV, TRM, OS, NRM, LFS, relapse, overall infections, and EBV reactivation do not seem to be affected by the type of utilised rATG. However, data on aGvHD grades III–IV, GRFS, moderate–severe cGvHD, and CMV reactivation is conflicting. Through our research, we sought to summarise the most recent findings concerning r-ATGs in allo-HCT, and provide insight into the differences between the targets and origin of various ATG formulations.

## 1. Introduction

GvHD stands as a paramount allo-HCT complication [1,2], as it detrimentally impacts both the duration and quality of life for post-transplant patients [3]. Elevated T cell count, HLA mismatch, and the employment of peripheral blood stem cells (PBSC) as a major transplant material nowadays represent factors of vulnerability for both aGvHD and cGvHD [4,5,6,7]. In spite of the administration of calcineurin inhibitors (CNIs) in conjunction with methotrexate (MTX) as prophylaxis of GvHD, numerous patients, ranging from 30% to 50%, develop aGvHD [8], while cGvHD persists in 30% to 70% of cases [9]. Hence, emphasis is put on developing appropriate immunosuppressive strategies that will not negatively affect the post-transplantation course.

In Europe, the prevailing therapeutic approach to prevent GvHD includes standard prophylaxis comprising CNIs, MTX, or mycophenolate mofetil in conjunction with one of the available r-ATGs for unrelated donor transplantation, and in recent years—for sibling as well [10]. There are a number of ATG formulations available in different countries, which originate from either rabbits, horses, or pigs, and are generated by the inoculation of human cell lines or human thymocytes. Porcine ATG (p-ATG) and horse ATG (h-ATG), as far as European countries are concerned, are rather rarely utilised medicaments. The former is employed in cases of severe aplastic anaemia in China and India and, to a lesser extent, in the context of allo-HCT [11,12], while the latter is considered the first-line therapy for moderate–severe aplastic anaemia [13] and GvHD prophylaxis [14].

There are currently two types of rATGs, which consist of polyclonal IgG obtained from the hyperimmune sera of rabbits. These IgG antibodies are immunised either with human thymocytes in the case of ATG-T (anti-thymocyte globulin, Thymoglobulin; Sanofi, Paris, France; formerly Genzyme), or with human Jurkat leukaemia T-cell lines in the case of ATG-G (anti-T-lymphocyte globulin, Grafalon; Neovii, Raperswil, Switzerland; formerly Fresenius) [15]. Furthermore, ATG-T and ATG-G differ also in the antigens to which they bind. ATG-T targets antigens expressed on T cells (CD2, CD3, CD4, CD6, CD8), B cells, natural killer cells, macrophages, and dendritic cells, HLA class 1 and HLA-DR [16]. ATG-T also contains antibodies that specifically target antigens associated with cellular adhesion and trafficking, along with antigens implicated in inflammation, apoptosis, and cellular proliferation [16]. The range of antigens recognised by ATG-G is narrower in comparison to that of ATG-T, as ATG-G contains few or no antibodies targeting CD3, CD4, or HLA-DR [17,18]. However, ATG-G has more antibodies directed against CD107, an antigen expressed on T cells during degranulation following antigenic stimulation [18]. Competitive binding experiments have revealed that ATG-T presents higher reactivity and a more potent complement-mediated cytotoxic effect towards peripheral blood mononuclear cells than ATG-G, and more effectively induces apoptosis of dendritic cells compared to ATG-G when equal doses of the two formulations are used. Hence, higher doses of ATG-G are administered in GvHD prophylaxis than ATG-T, as demonstrated in Table 1. The immunological consequences of ATG are also influenced by various factors, including the cumulative dosage, timing of administration in relation to allo-HCT, and the lymphocyte count of the recipient at the time of the transplantation. Higher doses of ATG, closer timing to transplantation, and lower host total lymphocyte count can result in prolonged exposure to ATG following the infusion of donor T cells [4]. This, in turn, delays immune reconstitution [19,20], thus increasing the potential for relapse, susceptibility to infections, and the development of post-transplant lymphoproliferative disorders [21]. Consequently, these factors are to be considered when assessing the outcomes when administering ATG.

According to the recommendations from an expert panel by Bonifazi et al. [21], ATG-T and ATG-G are strongly recommended as part of a myeloablative conditioning (MAC) regimen prior to bone marrow (BM) and PBSC allo-HCT from a matched or mismatched unrelated donor (MUD/MMUD), as prophylaxis of aGvHD and cGvHD. With limited evidence, ATG-T and ATG-G are also recommended prior to matched related donor (MRD) PBSC allo-HCT. In instances of reduced intensity or nonmyeloablative conditioning (RIC/NMA) regimens, being aware of a higher risk of relapse, ATG-T, and ATG-G are also efficacious in preventing aGvHD and cGvHD. Studies have also shown that ATG can effectively reduce the occurrence of GvHD and prolong the survival of patients who have undergone allo-HCT from unrelated donors (URDs) and haploidentical donors, without increasing relapse rates [22,23].

## 2. Materials and Methods

### 2.1. Systematic Literature Review

We performed a systematic literature review through PubMed, Scopus, Cochrane, and MEDLINE, searching both separately and individually variants of the following keywords: anti-thymocyte globulin, acute graft-versus-host disease, chronic graft-versus-host disease, allogeneic stem cell transplantation, Thymoglobulin, Grafalon (Supplementary Materials). Moreover, we analysed the references of various meta-analyses, reviews and studies. The search was conducted from 1 January 2017 to 14 June 2023, since the last article we retrieved comparing different r-ATGs in GvHD prophylaxis dates back to 2017 [24]. Studies were included that addressed r-ATG formulations in the context of GvHD prophylaxis and, additionally, reported data on overall survival (OS), transplantation-related mortality (TRM), non-relapse mortality (NRM), graft-versus-host/relapse-free survival (GRFS), leukaemia-free survival (LFS), relapse, and reactivations of infections, including CMV and EBV. The titles and abstracts were screened first, followed by the full text. Citations were excluded for the following reasons: economic outcomes, study phase, intervention, disease, design of the study, patient population, non-English.

### 2.2. Data Presentation, Extraction and Endpoints

**Figure 1:**
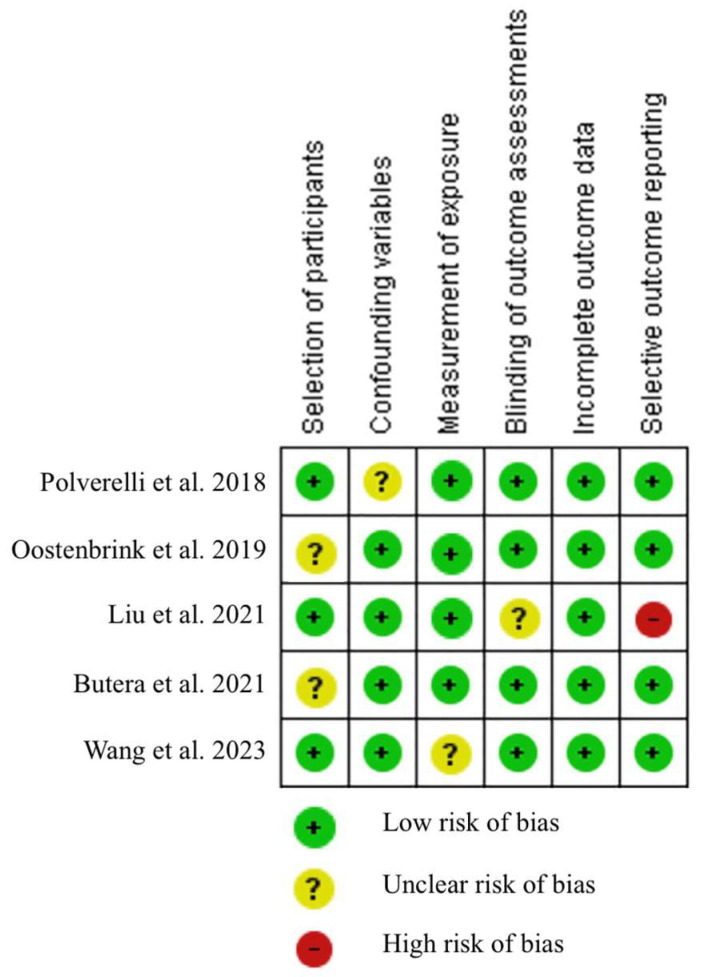
Summary of Risk of Bias Assessment tool for Non-randomized Studies (RoBANS) [25,26,27,28,29].

All the available data from the studies reporting on rates, hazard ratios (HRs) with or without 95% confidence intervals (CIs) was extracted, following the endpoints: cGvHD (all grades), aGvHD grade II–IV, aGvHD grade III–IV, OS, TRM, NRM, GRFS, LFS, relapse, and infection reactivations. Not all of the endpoints were discussed in each article. We present the details about each study in Figure 1, while each outcome, if reported, is noted in Table 2 and Table 3.

### 2.3. Risk of Bias Assessment

We evaluated the eligible studies identified during the study selection process, as delineated above, to determine their methodological quality and risk of bias. The quality assessment heavily relied on details regarding the trial’s design, implementation, data analysis, and outcome reporting. A validity assessment form, comprising the following elements as recommended by the Risk of Bias Assessment tool for Non-randomized Studies (RoBANS), was employed to evaluate quality and potential for bias: selection of participants, incomplete outcome data, confounding variables, measurement of exposure, blinding of outcome assessments, selective outcome reporting. The summary of the risk of bias assessment is presented in Figure 1.

## 3. Results

### Results—Systematic Literature Review

**Figure 2:**
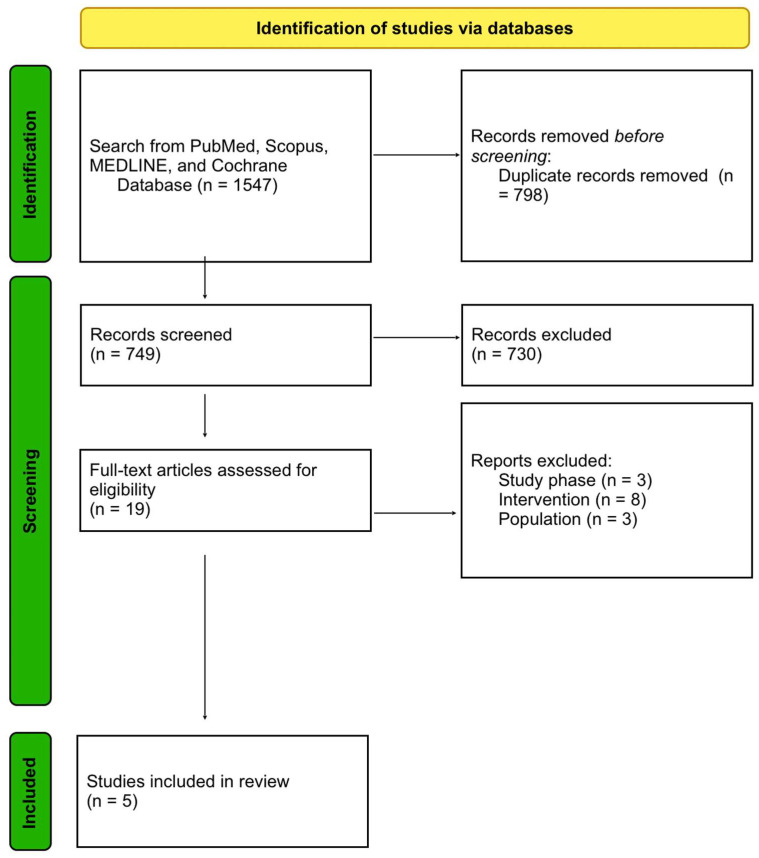
PRISMA flow diagram of the study selection process.

We retrieved a total of 1547 citations from the aforementioned databases. After removing duplicates 749 citations were left, and after a screening process considering titles and abstracts we excluded 730 articles from further analysis. In the next phase, 19 full texts were screened, of which five have been included in our review article. The flowchart of the identification of studies has been shown in Figure 2.

The citations consist of five full-text retrospective analyses published between 2017 and 2023, four of which carried out a comprehensive comparison of the effectiveness of ATG-T (anti-thymocyte globulin, Thymoglobulin; Sanofi, Paris, France; formerly Genzyme) and ATG-G (anti-T-lymphocyte globulin, Grafalon; Neovii, Raperswil, Switzerland; formerly Fresenius) [25,26,27,28], and one compared the outcomes of different ATG-T doses [29]. The overall population comprised 783 patients (ATG-T, *n* = 591; ATG-G, *n* = 192). Note that one of the articles’ population was paediatric [26].

## 4. Outcomes

In terms of overall cGvHD, aGvHD grades II–IV, TRM, OS, NRM, LFS, relapse, overall infections, and EBV reactivation, none of the included studies reported differences between ATG-T and ATG-G. There are discrepancies concerning the occurrence of aGvHD grades III–IV, as one of the studies revealed a significantly lower incidence when utilising ATG-G vs ATG-T (0% vs. 12%, *p* = 0.025) [26], while another has shown the opposite, with ATG-T being more effective in comparison to ATG-G (2.27% vs. 17.39%, *p* = 0.026) [27]. As for GRFS, one of the studies has confirmed the efficacy of instituting ATG-G vs ATG-T (67.4% vs. 41.9%, *p* = 0.042) [25], and low-dose ATG-T seems to lead to a longer GRFS compared to ATG-G (43.1% vs. 32.4%, *p* = 0.014) [29]. A major reduction in CMV reactivations has been observed in one study, with ATG-G causing substantially less of these than ATG-T (29.9% vs. 64.6%, *p* < 0.001) [28].

## 5. Discussion

This systematic review addressing the efficacy of various r-ATG formulations in the context of allo-HCT included five retrospective studies published between 2017 and 2023 with a total of 783 participants. Our main findings upon an in-depth analysis of these are discrepant results concerning more severe forms of aGvHD (grades III–IV), GRFS, and CMV reactivation. Changes in overall cGvHD, aGvHD grades II–IV, TRM, OS, NRM, LFS, relapse, overall infections, and EBV reactivation were statistically insignificant.

In spite of a serious progress in transplantation procedures, GvHD remains one of the major and most severe complications following allo-HCT [1,2], negatively affecting the patient’s quality of life and, in more advanced grades, GvHD may prove fatal [30]. Hence, an effort is made to find the most effective GvHD prophylaxis regimen, which would limit the occurrence of GvHD, while maintaining a satisfactory graft-versus-leukaemia (GvL) effect and low incidence of fatal infection reactivations. It is important, especially in patients undergoing allo-HCT from MMRD or MMUD with PBSC, which are well-known risk factors for GvHD [4,6]. ATG is a commonly employed approach for in vivo depletion of T cells, aiming to mitigate the occurrence of GvHD in patients undergoing HLA-matched or HLA-mismatched allo-HCT [31]. While there have been numerous studies confirming the feasibility of ATG in GvHD prophylaxis [32,33,34,35,36,37], very few discussed the impact of different r-ATG formulations.

Recently, four meta-analyses discussing the efficacy of ATG-T and ATG-G have been published [24,31,37,38]. None of them found any differences in terms of OS and NRM regardless of the rATG type in both related and unrelated donor settings [24,31,37,38]. Furthermore, Kumar et al. performed a subgroup analysis of OS and NRM according to ATG-T doses of <6 mg/kg and >6 mg/kg total, indicating no major impact of ATG-T doses on OS and NRM [31]. Soiffer et al. [39] addressed the efficacy of ATG-G in patients with haematological malignancies in an ATG-G vs placebo in MUD allo-HCT settings. The obtained results suggest that the use of ATG-G does not affect OS [39]. Similarly, Kroger et al. [34] conducted a study comparing ATG-G vs. no ATG-G in MRD allo-HCT, which implied the lack of ATG-G influence on OS. These outcomes are in line with what we discovered through an analysis of the articles included in Figure 1 and Table 2, with no major impact of neither ATG-T nor ATG-G on OS and NRM [25,26,27,28,29]. As for TRM, the outcomes of both our analysis of articles [25,26,27,28,29] and the network meta-analysis by Gagelmann et al. [24] are conforming, suggesting a comparable influence of ATG-G/ATG-T on TRM.

In terms of aGvHD and cGvHD, the aforementioned four meta-analyses on ATG-T/ATG-G found a significant reduction of GvHD incidence regardless of the instituted rATG formulation [24,31,37,38]. However, a network meta-analysis by Gagelmann et al. [24] indicated a higher efficacy of ATG-G in preventing cGvHD and aGvHD compared to ATG-T and standard treatment. Numerous studies suggest the effectiveness of both ATG-T and ATG-G in GvHD prophylaxis independently of the donor setting, as articles have already been published indicating their effectiveness in haploidentical allo-HCT [40,41,42], MRD allo-HCT [21], and MUD/MMUD allo-HCT [21]. Our review unveiled contrasting results concerning severe forms of aGvHD (grades III–IV), where one study revealed a higher effectiveness of ATG-T vs. ATG-G (2.27% vs. 17.39%, *p* = 0.026) [27], while another proved ATG-G to be more efficacious compared to ATG-T (0% vs. 12%, *p* = 0.025) [26]. Overall cGvHD and aGvHD grades II–IV did not seem to be affected by the utilised type of rATG [25,26,27,28,29]. The study by Polverelli et al. [25] revealed an interesting finding concerning GvHD prophylaxis comparing ATG-T vs. ATG-G in MUD allo-HCT. Despite finding no statistically significant differences between the two rATGs in terms of aGvHD and overall cGvHD, a reduced moderate–severe cGVHD occurrence was noticed in the ATG-G group (20% vs. 75%, *p* = 0.05) [25]. Moreover, in a MUD setting, a longer GRFS has been linked to ATG-G compared to ATG-T (67.4% vs. 41.9%, *p* = 0.042) [25]. In a different study comparing low-dose and high-dose ATG-T in MUD allo-HCT, a better GRFS has been associated with low-dose ATG-T rather than high-dose ATG-T in MUD allo-HCT (43.1% vs. 32.4%, *p* = 0.014) [29]. There are discrepancies about relapse, as contrary to the findings of both our review [25,26,27,28,29] and other researchers’ meta-analyses [30,32,39], Kumar et al. [31] reported a higher risk of disease relapse when employing ATG-T/ATG-G.

ATG-T and ATG-G target various antigens expressed on the surface of immune cells, with the spectrum of the former being much broader, resulting in a significantly stronger T-cell-depleting effect [16,17,18]. The administration of ATG-G and ATG-T, by inducing a delayed immune reconstitution [19,20], potentially elevates the risk of infections [43,44]. Due to divergent outcomes resulting from variations in rATG dosage, there is an ongoing debate about how strong of an impact it has on infection reactivations [21]. Despite there not being a consensus on this matter, most studies corroborate an overall tendency towards an increased risk of infection in patients subjected to higher rATG doses [45,46,47]. According to the studies we analysed in our review [25,26,27,28,29], neither the type of utilised rATG nor the doses have a significant influence on EBV reactivation and the overall occurrence of infections. As for CMV reactivation, most of the articles did not show any differences between ATG-T/ATG-G [25,26,27] and doses of ATG-T [29]. Interestingly, there has been a recent report that demonstrated a substantial difference in CMV reactivation in favour of ATG-G compared to ATG-T in an URD setting (29.9% vs. 64.6%, *p* < 0.001) [28]. The stronger immunosuppressive effect of ATG-T administered at a dosage of 10 mg/kg in contrast to ATG-G at a dosage of 20 mg/kg may be a potential explanation for this result [28]. In support of this hypothesis, two studies provided evidence of a connection between ATG-T at 10 mg/kg and a delayed T-cell reconstitution in comparison to ATG-G when utilised in doses of 25 mg/kg to 45–60 mg/kg [26,48]. Nowadays, while letermovir is a widely employed CMV prophylaxis in seropositive allo-HCT recipients, further research is mandatory in order to establish the most appropriate approach.

This article also has some limitations. First, it does not comprise a network meta-analysis and methodological quality assessment. Second, all of the included studies are retrospective with, in some instances, not very large sample sizes, and the distribution of baseline characteristics of patients may have been uneven. However, the overall risk of bias of the included studies was judged to be moderate. Thus, the data collected in our study might be inadequate to establish the robustness of the conclusions. To validate these findings, there is still a requirement for multicentre, large-scale, prospective, randomised controlled trials.

In conclusion, the type of utilised rATG does not seem to affect overall cGvHD, aGvHD grades II–IV, TRM, OS, NRM, LFS, relapse, overall infections and EBV reactivation. However, we have found conflicting results in what concerns aGvHD grades III–IV, moderate–severe cGvHD, GRFS, and CMV reactivations. In addition to the most adequate rATG formulation, the optimal timing, dosage and blood concentration of rATG are yet to be determined.

## Figures and Tables

**Table 1:** Types of ATG and summary of their main characteristics. Abbreviations: h-ATG—horse ATG, ATG-T—Thymoglobuline, Genzyme, ATG-G—Grafalon, Fresenius. Based on the chart by Baron et al. [15].

ATG Formulation	Type of Antibodies	Recommended Dose for GvHD Prophylaxis (Total, mg/kg)
h-ATG	Polyclonal IgG from horses immunised with human thymocytes	**-**
ATG-T	Polyclonal IgG from rabbits immunised with human thymocytes	2.5–10
ATG-G	Polyclonal IgG from rabbits immunised with human Jurkat T leukaemia cell line	15–60

**Table 2:** Endpoints with reported and extracted data. Abbreviations: ATG-T—Thymoglobulin, Genzyme, ATG-G—Grafalon, Fresenius, BM—bone marrow, PBSC—peripheral blood stem cells, AML—acute myeloid leukaemia, CML—chronic myeloid leukaemia, ALL—acute lymphoblastic leukaemia, MDS—myelodysplastic syndrome, MPNs—myeloproliferative neoplasms, ALAL—acute leukaemia with ambiguous lineage, FLU—fludarabine, BU—busulfan, CY—cyclophosphamide, MUD—matched unrelated donor, MRD—matched related donor, MMRD—mismatched related donor, MMUD—mismatched unrelated donor, URD—unrelated donor, Haplo—haploidentical donor, NR—not reported.

	Polverelli et al., 2018, [25], (*n* = 77)		Oostenbrink et al., 2019, [26], (*n* = 58)		Liu et al., 2021, [27], (*n* = 214–Total, *n* = 67–Selected for ATG-T, ATG-G) *		Butera et al., 2021, [29], (*n* = 395)		Wang et al., 2023, [28], (*n* = 186)	
Type of ATG utilised	ATG-T	ATG-G	ATG-T	ATG-G	ATG-T	ATG-G	ATG-T	ATG-T	ATG-G	ATG-T
Number of patients	*n* = 31 (40%)	*n* = 46 (60%)	*n* = 42 (72%) High-dose *n* = 24, Low-dose *n* = 18	*n* = 16 (28%) High-dose *n* = 9 Low-dose *n* = 7	*n* = 44 (66%)	*n* = 23 (34%)	*n* = 197 (50%)	*n* = 198 (50%)	*n* = 107 (58%)	*n* = 79 (42%)
Age (years), median (range)	45 (17–61)	48 (18–66)	9 (1–18)	6 (1–17)	27 (6–50)	26 (3–52)	52.4 (20.7–69.4)	50.4 (20.7–66.8)	25 (3–59)	30 (3–65)
Sex, (%) Male Female	*n* = 23 (74%) *n* = 8 (26%)	*n* = 29 (63%) *n* = 19 (37%)	NR	NR	*n* = 27 (61.36%) *n* = 17 (38.64%)	*n* = 13 (56.52%) *n* = 10 (43.48%)	*n* = 99 (50%) *n* = 98 (50%)	*n* = 117 (59%) *n* = 81 (41%)	*n* = 63 (58.9%) *n* = 44 (41.1%)	*n* = 50 (63.3%) *n* = 29 (36.7%)
Dose of ATG (total, mg/kg)	7.5 mg/kg	30 mg/kg	High-dose 10 mg/kg Low-dose 6–8 mg/kg	High-dose 60 mg/kg Low-dose 45 mg/kg	MRD 12.5 mg/kg Haplo 10 mg/kg	MRD 25 mg/kg Haplo 20 mg/kg	5 mg/kg	6–7.5 mg/kg	20 mg/kg	10 mg/kg
Follow-up (days/months), median (range)	20 (1–88) months	22 (2–60) months	NR	NR	47.65 (0.50–186.78) months	44.34 (3.0–76.15) months	81.5 (50.2–119.3) months	81.5 (50.2–119.3) months	NR	NR
Diagnosis	Acute leukaemia *n* = 17 (56%) MDS *n* = 1 (3%) MPNs *n* = 1 (3%) Lymphoproliferative neoplasms *n* = 11 (35%) Others *n* = 1 (3%)	Acute leukaemia *n* = 24 (52%) MDS *n* = 7 (15%) MPNs *n* = 2 (5%) Lymphoproliferative neoplasms *n* = 12 (26%) Others *n* = 1 (2%)	ALL *n* = 17 (40%) AML *n* = 25 (60%)	ALL *n* = 16 (100%)	Severe aplastic anaemia	Severe aplastic anaemia	ALL *n* = 23 (11.7%) AML/MDS *n* = 111 (56.3%) MPN *n* = 14 (7.1%) LPD *n* = 49 (24.9%)	ALL *n* = 29 (14.7%) AML/MDS *n* = 88 (44.4%) MPN *n* = 19 (9.6%) LPD *n* = 62 (31.3%)	ALAL *n* = 4 (3.7%) ALL *n* = 29 (27.1%) AML *n* = 42 (39.3%) CLL *n* = 1 (0.9%) CML *n* = 23 (21.5%) MDS *n* = 7 (6.5%) NHL *n* = 1 (0.9%)	ALAL *n* = 4 (5.1%) ALL *n* = 16 (20.3%) AML *n* = 43 (54.4%) CLL *n* = 0 (0%) CML *n* = 6 (7.6%) MDS *n* = 6 (7.6%) NHL *n* = 4 (5.1%)
Conditioning regimen	MAC *n* = 16 (52%) RIC *n* = 15 (48%)	MAC *n* = 22 (48%) RIC *n* = 24 (52%)	NR	NR	FLU + CY ^5^ *n* = 15 (34.01%) BU + CY ^5^ *n* = 29 (65.91%)	FLU + CY ^5^ *n* = 4 (17.39%) BU + CY ^5^ *n* = 19 (82.61%)	MAC *n* = 154 (78.2%) RIC *n* = 43 (21.8%)	MAC *n* = 107 (54%) RIC *n* = 91 (46%)	TBI/CY ^1^ *n* = 10 (9.3%) BU/CY ^2^ *n* = 60 (56.1%) Haplo ^3^ *n* = 30 (28.0%) FB3 ^4^ *n* = 6 (5.6%) Other *n* = 1 (0.9%)	TBI/CY ^1^ *n* = 3 (3.8%) BU/CY ^2^ *n* = 3 (3.8%) Haplo ^3^ *n* = 21 (26.6%) FB3 ^4^ *n* = 6 (7.6%) Other *n* = 0 (0%)
Stem cell source, (%) BM PBSC	BM *n* = 5 (16%) PBSC *n* = 26 (84%)	BM *n* = 5 (11%) PBSC *n* = 41 (89%)	BM *n* = 34 (81%) PBSC *n* = 8 (19%)	BM *n* = 14 (87%) PBSC *n* = 2 (13%)	BM + PBSC *n* = 28 (63.64%) BM *n* = 10 (22.73%) PBSC *n* = 6 (13.64%)	BM + PBSC *n* = 18 (78.26%) BM *n* = 2 (8.7%) PBSC *n* = 3 (13.04%)	BM *n* = 25 (12.7%) PBSC *n* = 172 (87.3%)	BM *n* = 30 (15.15%) PBSC *n* = 168 (84.85%)	NR	NR
Donor	MUD	MUD	MUD *n* = 30 (71%) MMUD *n* = 12 (29%)	MUD *n* = 13 (81%) MMUD *n* = 3 (19%)	MRD *n* = 13 (29.55%) Haplo *n* = 28 (63.64%) URD *n* = 3 (6.82%)	MRD *n* = 6 (26.09%) Haplo *n* = 16 (69.57%) URD *n* = 1 (4.35%)	MUD	MUD	MUD *n* = 69 (64.5%) MMUD *n* = 38 (35.5%)	MUD *n* = 45 (57.0%) MMUD *n* = 34 (43.0%)

* Of the initially enrolled 214 patients, only 67 were selected after propensity score matching. ^1^ 8–9.5 Gy total body irradiation was delivered and fractioned by two days, and a total dose of 120 mg/kg cyclophosphamide was administered. ^2^ A total dose of 12.8 mg/kg intravenous busulfan and 120 mg/kg cyclophosphamide was administered. ^3^ CCNU/MECCNU + Ara-c + BU + CY − 200 mg/m^2^ lomustine or semustine, a total dose of 8 g/m^2^ cytarabine, 9.6 mg/kg intravenous busulfan and 3.6 g was administered. This regimen is usually used in Haplo-HCT in China. ^4^ A total dose of 150 mg/m^2^ fludarabine and 390 mg/m^2^ busulfan was administered. ^5^ Doses were not reported.

**Table 3:** Reported and extracted endpoints. Abbreviations: ATG-T—Thymoglobulin, Genzyme, ATG-G—Grafalon, Fresenius, OS—overall survival, TRM—transplantation-related mortality, NRM—non-relapse mortality, GRFS—graft-versus-host/relapse-free survival, LFS—leukaemia-free survival, NR—not reported.

Endpoint	Polverelli et al., 2018 [25], (*n* = 77)		Oostenbrink et al., 2019, [26], (*n* = 58)		Liu et al., 2021, [27], (*n* = 214–Total, *n* = 67–Selected for ATG-T, ATG-G) *		Butera et al., 2021, [29], (*n* = 395)		Wang et al., 2023, [28], (*n* = 186)	
Type of ATG	ATG-T	ATG-G	ATG-T	ATG-G	ATG-T	ATG-G	ATG-T (5 mg/kg total)	ATG-T (6–7.5 mg/kg total)	ATG-G	ATG-T
Chronic GvHD	*n* = 8 (31%) *p* = 0.77	*n* = 10 (26%) *p* = 0.77	High-dose *n* = 6 (25%) Low-dose *n* = 3 (17%) *p* = 0.97	*n* = 2 (13%) *p* = 0.97	26.83% *p* = 0.704	22.73% *p* = 0.704	Moderate-severe cGvHD 17.4% *p* = 0.34	Moderate-severe cGvHD 20.3% *p* = 0.34	43.9% *p* = 0.279	28.8% *p* = 0.279
Acute GvHD grade II–IV	*n* = 13 (42%)	*n* = 20 (43%)	High-dose *n* = 2 (8%) Low-dose *n* = 6 (33%)	*n* = 6 (38%)	20.45% *p* = 0.948	21.74% *p* = 0.948	28.6% *p* = 0.18	33.9% *p* = 0.18	8.4% *p* = 0.583	6.3% *p* = 0.583
Acute GvHD grade III–IV	*n* = 3 (10%) *p* = 0.39	*n* = 2 (4%) *p* = 0.39	**High-dose** ***n* = 1 (4%)** **Low-dose** ***n* = 4 (22%)** ***p* = 0.025**	***n*** **= 0 (0%)** ***p* = 0.025**	**2.27%** ***p* = 0.026**	**17.39%** ***p* = 0.026**	10.2% *p* = 0.26	13.7% *p* = 0.26	NR	NR
OS	5-years period *n* = 35 (43%) *p* = 0.58		High-dose 62 months (1–92) Low-dose 33 months (4–53) *p* = 0.15	34 months (4–84) *p* = 0.15	5-year period 86.4% *p* = 0.245	5-year period 95.7% *p* = 0.245	56.6% *p* = 0.052	46.3% *p* = 0.052	75% *p* = 0.645	80.9% *p* = 0.645
TRM	5 years period *n* = 18 (24.5%) *p* = 0.54		High-dose *n* = 1 Low-dose *n* = 0	*n* = 0	11.36% *p* = 0.614	4.35% *p* = 0.614	NR	NR	NR	NR
NRM	5 years period *n* = 19 (25.65%) 45%		NR	NR	NR	NR	5-year period 27.9% *p* = 0.094	5-year period 21.5% *p* = 0.094	10.4% *p* = 0.402	15% *p* = 0.402
GRFS	**2 years period** **41.9% ** ***p* = 0.042**	**2 years period** **67.4%** ***p* = 0.042**	NR	NR	GVHD-free, failure-free survival 77.3% *p* = 0.986	GVHD-free, failure-free survival 78.3% *p* = 0.986	**43.1%** ***p* = 0.014**	**32.4%** ***p* = 0.014**	33.5% *p* = 0.109	52.8% *p* = 0.109
LFS	NR	NR	NR	NR	NR	NR	46.3% *p* = 0.051	38.6% *p* = 0.051	NR	NR
Relapse	2 years period 32% *p* = 0.41	2 years period 38% *p* = 0.41	High-dose *n* = 4 (16%) Low-dose *n* = 4 (22%) *p* = 0.54	*n* = 3 (18%) *p* = 0.54	NR	NR	5-year period 31.7% *p* = 0.66	5-year period 33.6% *p* = 0.66	33.5% *p* = 0.153	19.4% *p* = 0.153
CMV reactivation	*n* = 22 (71%) *p* = 0.23	*n* = 26 (57%) *p* = 0.23	High-dose *n* = 5 Low-dose *n* = 7 *p* = 0.62	*n* = 4 *p* = 0.62	NR	NR	Day 100 32.7% *p* = 0.3	Day 100 35.6% *p* = 0.3	**29.9%** ***p* < 0.001**	**64.6%** ***p* < 0.001**
EBV reactivation	NR	NR	High-dose *n* = 7 Low-dose *n* = 4 *p* = 0.28	*n* = 2 *p* = 0.28	NR	NR	10.7% *p* = 0.95	11.1% *p* = 0.95	NR	NR
Infections overall	*n* = 30 (97%) *p* = 1	*n* = 45 (98%) *p* = 1	NR	NR	59.09% *p* = 0.84	56.52% *p* = 0.84	NR	NR	NR	NR

* Of the initially enrolled 214 patients, only 67 were selected after propensity score matching.

## Data Availability

Not applicable.

## Supplementary Materials

The following supporting information can be downloaded at: https://www.mdpi.com/article/10.3390/jcm12175449/s1, File S1: PRISMA-P (Preferred Reporting Items for Systematic review and Meta-Analysis Protocols) 2015 checklist: recommended items to address in a systematic review protocol. Reference [49] is cited in the supplementary materials.

## Abbreviations

The following abbreviations have been used in this manuscript:

AML	acute myeloid leukaemia
ALAL	acute leukaemia with ambiguous lineage
ALL	acute lymphoblastic leukaemia
allo-HCT	allogeneic hematopoietic stem cell transplantation
ATG-G	Grafalon
ATG-T	Thymoglobulin
h-ATG	horse ATG
p-ATG	porcine ATG
BM	bone marrow
BU	busulfan
CIs	confidence intervals
CML	chronic myeloid leukaemia
CMV	cytomegalovirus
CNIs	calcineurin inhibitors
CY	cyclophosphamide
EBV	Epstein-Barr virus
FLU	fludarabine
GRFS	graft-versus-host/relapse-free survival
GvHD	graft-versus-host disease
aGvHD	acute graft-versus-host disease
cGvHD	chronic graft-versus-host disease
GvL	graft-versus-leukaemia
Haplo	haploidentical donor
HRs	hazard ratios
LFS	leukaemia-free survival
MAC	myeloablative conditioning
MDS	myelodysplastic syndrome
MRD	matched related donor
MMRD	mismatched related donor
MUD	matched unrelated donor
MMUD	mismatched unrelated donor
MPNs	myeloproliferative neoplasms
MTX	methotrexate
NMA	nonmyeloablative conditioning
NR	not reported
NRM	non-relapse mortality
OS	overall survival
PBSC	peripheral blood stem cells
r-ATG	rabbit anti-thymocyte globulin
RCTs	randomised controlled trials
RIC	reduced intensity conditioning
TRM	transplantation-related mortality
URD	unrelated donor
